# New Perspectives in Endoscopic Treatment of Gastroesophageal Reflux Disease

**DOI:** 10.3390/diagnostics13122057

**Published:** 2023-06-14

**Authors:** Federica Calabrese, Valeria Poletti, Francesco Auriemma, Danilo Paduano, Carmine Gentile, Antonio Facciorusso, Gianluca Franchellucci, Alessandro De Marco, Luca Brandaleone, Andrew Ofosu, Jayanta Samanta, Daryl Ramai, Luca De Luca, Abed Al-Lehibi, Walter Zuliani, Cesare Hassan, Alessandro Repici, Benedetto Mangiavillano

**Affiliations:** 1 Gastrointestinal Endoscopy Unit, Humanitas Mater Domini, 21053 Castellanza, Italy; 2 Endoscopy Unit, Humanitas Clinical and Research Center—IRCCS, 20089 Rozzano, Italy; 3 Section of Gastroenterology, Department of Medical Sciences, University of Foggia, 71100 Foggia, Italy; 4 Division of Digestive Diseases, University of Cincinnati, Cincinnati, OH 45219, USA; 5 Department of Gastroenterology, Post Graduate Institute of Medical Education and Research, Chandigarh 160012, India; 6 Division of Gastroenterology and Hepatology, University of Utah Health, Salt Lake City, UT 84132, USA; 7 Endoscopic Unit, ASST Santi Paolo e Carlo, 20142 Milan, Italy; 8 King Fahad Medical City, Faculty of Medicine, King Saud Bin Abduaziz University-Health Science, Riyadh 11525, Saudi Arabia; 9 Department of Biomedical Sciences, Humanitas University, 20072 Pieve Emanuele, Italy

**Keywords:** gastroesophageal reflux disease (GERD), transoral incisionless fundoplication (TIF), anti-reflux mucosal interventions (ARMI), anti-reflux mucosal resection (ARMS), anti-reflux mucosal ablation (ARMA)

## Abstract

Gastroesophageal reflux disease has a high incidence and prevalence in the general population. Clinical manifestations are heterogenous, and so is the response to medical treatment. Proton pump inhibitors are still the most common agents used to control reflux symptoms and for healing esophagitis, but they are not a one-size-fits-all solution for the disease. Patients with persistent troublesome symptoms despite medical therapy, those experiencing some adverse drug reaction, or those unwilling to take lifelong medications deserve valid alternatives. Anti-reflux Nissen fundoplication is an effective option, but the risk of adverse events has limited its spread. In recent years, advancements in therapeutic endoscopy have been made, and three major endoluminal alternatives are now available, including (1) the delivery of radiofrequency energy to the esophago–gastric junction, (2) transoral incisionless fundoplication (TIF), and (3) anti-reflux mucosal interventions (ARMI) based on mucosal resection (ARMS) and mucosal ablation (ARMA) techniques to remodel the cardia. Endoscopic techniques have shown interesting results, but their diffusion is still limited to expert endoscopists in tertiary centers. This review discusses the state of the art in the endoscopic approach to gastroesophageal reflux disease.

## 1. Introduction

Gastroesophageal reflux disease (GERD) is a common and increasing gastrointestinal disorder with an estimated 20% prevalence worldwide and a geographical trend towards Western countries. The last 30 years have seen an almost 80% increase in its prevalence, mainly attributed to population aging and changes in lifestyle and dietary habits linked to the obesity epidemic [1]. Its chronic and recurrent course notably impacts patients’ physical and mental health. Additionally, a relevant socio-economic burden follows with both direct and indirect costs [2,3].

GERD occurs as a consequence of the failure of the normal anti-reflux barriers to protect the esophageal mucosa against a reflux of gastric content, which leads to troublesome symptoms or local complications secondary to mucosal damage, as stated in the Montreal consensus of 2006 [4]. The lower esophageal sphincter (LES), along with the crural diaphragm, works as a “high-pressure zone” that prevents abnormal retrograde flow of gastric content into the esophagus. In healthy individuals, the LES and the diaphragmatic hiatus open synchronously in response to swallowing or belching: in this setting, GERD represents a physiological event and can happen many times per day. When dysfunction of this “high-pressure zone” occurs, transient complete relaxations of the LES (TLESRs) not initiated by swallowing can become frequent in the postprandial period and contribute to up to 90% of reflux episodes [5]. Major mechanisms involved in GERD pathophysiology also include mechanical incompetence of the LES due to a short length or a low resting pressure, impaired esophageal clearance, impaired mucosal barrier, and delayed gastric emptying. Adjunctive risk factors are the presence of hiatal hernia or an increase in intra-abdominal pressure as seen in obesity, bile acid reflux, excessive alcohol or caffeine consumption, smoking habit, and the use of certain medications such as nitrates, calcium channel blockers, antidepressants, benzodiazepines, and non-steroidal anti-inflammatory drugs [2].

Clinical manifestations of GERD are heterogeneous. The main presentation includes the “typical” esophageal symptoms of heartburn and regurgitation, variably accompanied by epigastric pain, dyspepsia, bloating, and dysphagia. A plethora of “atypical” manifestations are also acknowledged, defined as extraesophageal syndromes, including cardiac (e.g., non-cardiac chest pain, arrhythmias) and laryngopharyngeal or pulmonary symptoms (e.g., chronic cough, sore throat, hoarseness, globus sensation, asthma) [4]. 

The clinical history of typical symptoms combined with effective medical treatment response has traditionally been used as a cost-effective solution to formulate a presumptive diagnosis of GERD without further investigation [6,7]. Patient self-report on symptoms is believed to be more reliable than physician assessment. Many symptom-focused self-administered questionnaires have been validated over the years as a measure of response to treatment. These questionnaires play a more important role in clinical trials than in clinical practice, and the most often employed are the Gastro-esophageal Reflux Disease Health-Related Quality of Life (GERD-HRQL) [8,9], GERD Questionnaire (GERD-Q) [10], Frequency Scale for the Symptoms of GERD (FSSG or F-scale) [11], and Reflux Symptom Index (RSI) for laryngopharyngeal symptoms [12].

When the clinical presentation in GERD is atypical, or the response to acid suppression is insufficient, instrumental tests take on a role in the diagnosis, namely upper endoscopy and ambulatory function tests. Upper endoscopy must be offered in the presence of any red flag (e.g., age >50 years, dysphagia or odynophagia, unexplained anemia or manifest bleeding, unintentional weight loss, recurrent vomiting, positive family history of cancer) within a long course of disease and/or in the setting of extraesophageal syndromes after the exclusion of cardiovascular or pulmonary causes for symptoms. Endoscopy can show reflux signatures such as esophagitis graded according to the Los Angeles classification [13], Barrett’s esophagus, or peptic strictures. Based on the absence or presence of one or more of these findings, GERD has been categorized into non-erosive (NERD) or erosive disease, respectively [7]. High-resolution manometry (HRM) measures the esophago–gastric junction (EGJ) relaxation and, specifically, LES pressure; it can also demonstrate a spatial dissociation of the intrinsic LES and the diaphragm or rule out an abnormal esophageal motility pattern [14]. Combined 24 h pH impedance monitoring can demonstrate either abnormal esophageal acid exposure time (AET) or an abnormal number of refluxes, distinguishing acidic, weakly acidic, and weakly alkaline refluxes, and be used to evaluate the correlation between reflux episodes and patients’ symptoms (symptom index, SI, and symptom association probability, SAP) [6]. The application of impedance monitoring allowed the identification of two subgroups within the group of patients earlier classified as NERD: those having a normal AET but a positive reflux symptom association, defined as reflux hypersensitivity (RH), and those with no pathological AET nor positive symptom association, now defined as functional heartburn (FH), and who do not have GERD [15].

This review aims to discuss the principal endoscopic options introduced for GERD in recent years and envisage their possible role alongside the consolidated pharmacological and surgical treatments.

## 2. Methods

The PubMed/MEDLINE database was systematically searched for articles published in English between 1 January 2010 and 28 February 2023. Combinations of keywords based on the Medical Subject Headings (MeSH) were used, including “gastroesophageal reflux disease”, “transoral incisionless fundoplication”, “anti-reflux mucosectomy”, and “anti-reflux mucosal ablation”. Randomized controlled trials and metanalysis regarding endoscopic techniques adopted to treat GERD were considered eligible and included. Observational studies and large cohort retrospective series were also evaluated.

## 3. Medical Treatment

Lifestyle modifications and medical treatment are widely recognized as the initial strategy for GERD treatment, as they are effective for symptomatic relief and mucosal healing in a large proportion of patients. 

Weight loss and physical activity, good sleep hygiene, modifications of meal habits, and limited intake of ‘triggering’ foods are usually recommended to patients, despite controversial evidence [16,17]. 

Medical treatment focuses on using antisecretory drugs, including histamine receptor blockers (H2RAs), which act through the competitive inhibition of a histamine-stimulated acid secretion, and proton pump inhibitors (PPIs), which covalently bind and disable activated hydrogen potassium ATPase. A series of coadjuvant drugs are also available, including alginate-based antacid combinations, mucosal protective agents, and prokinetics. 

### 3.1. Antisecretory Drugs

H2RAs played a key role until the introduction of PPIs. They can suppress both basal and stimulated gastric acid secretion by reversibly binding to the histamine H2 receptors located on gastric parietal cells; the onset of action takes about 60 min, with a range of duration from 4 to 10 h. Marketed H2RAs include cimetidine, famotidine, and nizatidine; ranitidine has been withdrawn for carcinogen contamination during manufacturing. H2RAs are generally well tolerated, with mild side effects including headache, drowsiness, fatigue, abdominal pain, constipation, or diarrhea; central nervous system side effects such as delirium or confusion have been described in elderly patients with renal or hepatic impairment.

H2RAs are approved for short-term use (intermittent, or as needed) in uncomplicated GERD, both in monotherapy and in association with antacids or PPIs at a once-daily dose at nighttime to eliminate nocturnal acid breakthrough episodes. Tachyphylaxis or tolerance, generally occurring within 7 to 14 days of continued treatment, limits their use as maintenance therapy [18]. Over the past 30 years, PPIs (omeprazole, lansoprazole, rabeprazole, pantoprazole, and esomeprazole) have progressively become the cornerstone of GERD medical treatment, proving superior to H2RAs in terms of acid suppression. 

As they directly block the acid pump itself, acid secretion is inhibited for up to 36 h until replacement pumps can be synthesized; they are able to maintain intragastric pH > 4 for between 15 and 21 h daily, show superior efficacy in postprandial and nocturnal intragastric pH control, and can be used for long-term maintenance without the need for dose escalation. However, suboptimal dose timing may limit their efficacy because of a short serum half-life and the need for meal-induced ATPase activation; this leads to the necessity of preprandial dosing (about 30 to 60 min before a meal) and multiday treatment [18,19,20,21]. 

A 4- to 8-week course of PPIs at standard dose is highly effective in heartburn remission and mucosal healing in erosive esophagitis, with a success rate above 80%. Nevertheless, the maintenance of endoscopic remission lowers to about 65% at 12 months.

Mixed results are seen when focusing on symptoms. For patients with a full response to a first PPI course, tapering to the lowest dosage effective for symptom control is advisable for the long term. Additionally, a subset of patients can do well with intermittent or on-demand treatment, disproving the adage “once on a PPI, always on a PPI”. That being said, about one third of patients will still experience inadequate symptom control at some point, especially those with NERD or atypical symptoms [22,23].

Partial or complete failure with daily PPI therapy is referred to as refractory GERD. Many factors can contribute to a lack of response to PPIs, including an inadequate dosage or incorrect prescription, non-adherence to therapy, rapid drug metabolism, residual acid reflux due to inadequate acid suppression, nocturnal acid escape, or non-acid reflux. Nevertheless, the possibility of misdiagnosis should not be underestimated, considering FH in the first place [23,24]. Concurrently, in the last decade, a growing number of publications have warned about the potential adverse effects of long-term PPI therapies, such as the risk of small intestinal bacterial overgrowth (SIBO) and micronutrient malabsorption, intestinal infections including Clostridium difficile, osteoporosis-related fractures, kidney disease, dementia, cardiovascular events, and gastric cancer [25]. Even though no certain causality has been established, clinicians and patients have felt increasingly encouraged to investigate alternative non-drug therapeutic approaches. 

### 3.2. Antacids, Alginate, and Mucosal Protective Agents

Antacids are compounds containing sodium bicarbonate, aluminum hydroxide, magnesium hydroxide, magnesium carbonate, or calcium carbonate, which buffer excess gastric hydrochloric acid and inhibit pepsin activity; they offer rapid but temporary symptomatic relief. For a protracted effect, combinations of alginate and low-dose antacids are used; alginate is a natural polysaccharide polymer that acts as a mechanical barrier, forming a gel raft that displaces and neutralizes the post-prandial “acid pocket” in the proximal stomach. The latest developed formulations contain hyaluronic acid and chondroitin-sulphate on a bioadhesive carrier, which form a protective layer to favor mucosal hydration, healing, and regeneration [26,27,28]. 

Patients reporting mild to moderate intermittent reflux symptoms can consider alginate-based antacid combinations and mucosal protective agents as initial therapy because of their efficacy on demand, safety profile, and over-the-counter availability. These drugs are useful for relieving both heartburn and laryngopharyngeal symptoms; they can also have a role as an add-on to H2RAs or PPIs in patients with an incomplete response. Central to their efficacy is the ability to target reflux mechanisms not strictly related to the acid content, such as pepsin’s proteolytic activity, an impaired mucosal barrier, or visceral hypersensitivity [26,28,29]. 

Regarding prokinetics, such as metoclopramide, domperidone, or levosulpiride, there is no high-quality evidence to recommend their routine use in GERD. However, a benefit from their usage exists for patients with associated delayed gastric emptying or gastroparesis [30].

### 3.3. Potassium-Competitive Acid Blockers (PCABs)

The latest innovation in the medical management of GERD is represented by potassium-competitive acid blockers (PCABs), with the first-in-class named Vonoprazen. Their mechanism of action consists of preventing potassium ions from binding to gastric ATPase, providing reversible acid suppression. Vonoprazen has been approved in Japan since 2015 for treating GERD, peptic ulcer disease, and Helicobacter pylori infection. More recently, its safety and efficacy have been proved in PPI refractory GERD. Food and Drug Administration (FDA) approval came in 2022, but the drug is not marketed in Europe yet [31,32].

## 4. Surgery

Anti-reflux surgery has been traditionally offered as an alternative to medical treatment. It mainly relies on laparoscopic fundoplication (LARS), which consists of recreating a competent anti-esophageal reflux valve wrapping the gastric fundus around the distal esophagus, with the concurrent reconstruction of the diaphragmatic hiatus and hiatal hernia reduction if present. The Nissen technique involving a 360-degree total wrap has become the gold standard in anti-reflux surgery since its introduction in the early 1990s [33]. Partial fundoplications are also used, including the 270-degree posterior Toupet and the 180-degree anterior Dor fundoplication. While the Nissen approach guarantees better long-term durability, the partial wrap could reduce post-operative complaints [34,35].

The current consensus is to advise LARS in patients with typical or atypical symptoms responsive to PPIs desiring to discontinue therapy or who have developed side effects of medications, and/or in the presence of sliding hiatal hernia, Barrett’s esophagus, or esophagitis (Los Angeles grade B or higher) off PPI. Surgery is a reasonable option for regurgitation not controlled by medical therapy. Nevertheless, patients not responding to PPIs should be approached cautiously, as a wrong initial diagnosis may explain this. Patients with RH can also be favorable candidates for LARS, but those with FH should not be proposed [36]. A comprehensive diagnostic evaluation, including upper endoscopy, barium esophagogram, HRM, and 24 h pH impedance monitoring, must be carried out before LARS to confirm objective evidence of GERD, rule out non-GERD causes for PPI refractory symptoms, and exclude contraindications to surgery [37]. 

The success rates of LARS range from 67% to 95% but depend highly on surgical expertise and appropriate patient selection. Mortality is rare (<1%), and early complications primarily include infection (1.1%), bleeding (0.9%), and esophageal perforation (0.9%) [37,38].

A rate of new post-operative symptoms such as dysphagia, gas-bloat syndrome, or bowel dysfunction up to 25% has been described. Dysphagia is the most common complaint. Up to 50% of patients may experience early dysphagia related to post-surgical edema that usually improves within 3 months. Nevertheless, in 10% of cases, dysphagia is persistent, mainly attributable to a very tight or long fundoplication and less commonly to an underlying esophageal dysmotility. Gas-bloat syndrome may present with abdominal bloating, the inability to belch or vomit, postprandial fullness, nausea, flatulence, and epigastric pain. This complex of symptoms may be related to the impaired relaxation of the recreated flap valve in response to gastric distension and alteration in receptive gastric relaxation and accommodation. With a mechanism that is not well understood, about 18 to 33% of patients may have diarrhea [37,39].

Success rates in symptom control seem to reduce over time, mainly due to the progressive structural dysfunction of the fundoplication, such as hiatal herniation as a consequence of wrap disruption. A recent meta-analysis found that patients undergoing surgical treatment had superior short-term but not long-term (> 5 years follow-up) quality of life compared to medically treated patients. Symptom control was better with surgery than PPIs, even at long-term follow-up, but 28% of patients still used PPIs after LARS [40]. From long-term follow-up studies, 5 to 15 years after their anti-reflux surgery, 25% to 62% of patients are back on some type of acid-suppressive medication. When fundoplication fails, surgical revision should be reserved as a last-resort option and performed by experienced surgeons because redo surgery has increased morbidity and mortality compared with the primary operation, partly related to adhesion formation and altered anatomy [37,41].

## 5. Endoscopy

Endoluminal anti-reflux treatments have caught attention as minimally invasive options compared to classical surgical intervention for patients with minor anatomical impairment of the anti-reflux barrier (absence or very small hiatal hernia). The available techniques can be summarized in three groups: (1) delivery of radiofrequency energy to the EGJ, (2) endoscopic fundoplication, and (3) resective and ablative options.

### 5.1. Stretta

The first endoscopic treatment approved by the FDA for GERD is the Stretta System (Mederi Therapeutics, Norwalk, CT, USA). Radiofrequency energy is delivered to the muscularis propria 2 cm above and below the EGJ, applied at low frequency (465 MHz) and low power output (5W) with a temperature range of 65–85 °C. This setting for radiofrequency does not induce tissue ablation but presumably works by inducing hypertrophy of the LES muscle and thus reducing TLESRs and esophageal acid sensitivity [42]. Literature data support that Stretta treatment is safe, repeatable, and well tolerated, with a lasting effect over time. The Society of American Gastrointestinal and Endoscopic Surgeons (SAGES) guidelines for GERD, published in early 2013, gave this procedure a high level of evidence and strong recommendation only for patients with chronic GERD symptoms responsive to PPIs [43,44]. However, due to its low complication rate, Stretta is also considered in clinical practice for PPI refractory GERD, RH, and FH [42]. 

### 5.2. Endoscopic Fundoplication

Endoscopic fundoplication is a minimally invasive approach that mirrors LARS in terms of reconstructing a competent anti-reflux valve by fixing the gastric fundus around the lower esophagus. Throughout the past decades, many devices have been developed, but most have been withdrawn from the market because of concerns regarding safety and efficacy. Presently, the most popular systems include EsophyX (EndoGastric Solutions, Redmond, WA, USA), GERD-X (G-SURG GmbH, Seeon-Seebruck, Germany), and MUSE (Medigus, Omer, Israel).

#### 5.2.1. Transoral Incisionless Fundoplication (TIF)—EsophyX

The EsophyX device was first approved by the FDA in 2007. Over the years, the device and the procedure itself have undergone some modifications. The original procedure (TIF 1.0) essentially consisted of a short cardio-gastric plication. The transition to TIF 2.0 with the most recent version of the device, the EsophyX Z+, allows the recreation of a 3 to 5 cm long, 270-degree rotational wrap designed in an omega shape that is similar to what is obtained with LARS [45,46]. The procedure is performed under general anesthesia via endotracheal intubation, two operators are needed, and it takes about 40 min for the most experienced to complete the procedure. The EsophyX platform works as an overtube fitted over a standard gastroscope and is equipped with a vacuum suction system that stabilizes the lower esophagus through the diaphragm, if necessary, reducing small hiatal hernia. The procedure is standardized as follows: under full visualization from a retroflexed position in the stomach, six to eight non-resorbable polypropylene fasteners are deployed two at a time along the posterior wall first, and then along the anterior wall; an additional eight or more fasteners are deployed along the greater curvature to stabilize the length of the valve [47,48].

Since the fundoplication is partial and the esophageal lumen is controlled by the diameter of the device, overtightening is prevented [49]. For this reason, TIF is very rarely associated with dysphagia and virtually never causes gas-bloat syndrome.

Patients’ recovery is supposed to be faster than with LARS; usually, only a one-night stay in hospital is required. Common postoperative complications on day 1 have been described to be mild and transient, including sore throat, cough, belching, pain, nausea, vomiting, bloating, constipation, and left shoulder pain [50]. After the procedure, analgesics and antiemetics are usually administered for pain control and to prevent early vomiting, which can compromise the efficacy of the deployed fasteners. Patients are discharged with a short course of PPIs and on a liquid diet for a few weeks, then can gradually restart a regular diet. 

Post-marketing surveillance data from the FDA Manufacturer and User Facility Device Experience (MAUDE) database reported 131 complications out of a total of approximately 22,000 procedures over a 10-year period, including 26 cases of perforation (19.8%), 12 cases of bleeding (9.2%), 12 cases of pleural effusion (9.2%), 2 cases of pneumothorax (1.5%), 1 case of pulmonary embolism (0.8%), and 1 case of pneumomediastinum (0.8%). Complications were mostly treated conservatively or endoscopically, but in nine cases, emergent open surgery was needed [51]. 

According to a meta-analysis published in 2016 by Huang, including 4 RCTs and 12 prospective observational trials, the pooled rate of serious adverse events (SAEs) in patients undergoing TIF was 2.4%. It is important to note that this paper included data regardless of the version of the device used [52].

The ideal candidate for TIF presents with typical reflux symptoms responsive to PPIs, AET > 4%, and a Hill grade of I or II. TIF can also be reasonably performed in the presence of a small reducible hiatal hernia (<2 cm) or mild esophagitis (LA grade A and B) [42]. Interesting data have also become available for PPI non-responders and patients experiencing atypical or extraesophageal symptoms. 

Exclusion criteria are hiatal hernia >2 cm, esophagitis graded C or D according to the Los Angeles classification, Barrett’s esophagus >2 cm, and obesity class 2 onwards. The main reasons for treatment failure have proved to be inappropriate patient selection and especially an inaccurate identification of hiatal hernia.

The first multicentric prospective single-arm trial on EsophyX was conducted in 2010 on 100 consecutive patients suffering from GERD for over a year and with a history of daily PPI use for more than 6 months. TIF procedures were performed without complications. At the 6-month follow-up, GERD-HRQL was normalized in 73% of patients. Median heartburn, regurgitation, and RSI improved significantly, and 80% of patients stopped PPI usage completely [53].

In 2015, the TIF 2.0 EsophyX vs. Medical PPI Open label (TEMPO) multicentric trial was published, comparing the efficacy of TIF against PPIs [54]. Patients were randomly assigned to receive either TIF 2.0 or a maximum PPI dosage. After a 6-month observation, all patients in the PPI group were selected for crossover to the TIF procedure; therefore, all 63 patients underwent endoscopic treatment, and each patient served as their own control in comparing the efficacy of TIF against PPIs. No SAEs were registered, although three patients underwent reoperation by the end of the follow-up. Outcomes were recorded in follow-ups after 6 months, 3 years, and 5 years. Data at 6 months showed the superiority of TIF versus PPIs in terms of improving regurgitation (97% vs. 50%) and also, interestingly, in improving extra esophageal manifestations (62% vs. 5%). At 5 years, 44 patients were available for follow-up: 86% maintained a clinical response on regurgitation and 80% on atypical symptoms. In total, 10% of patients needed to resume daily PPIs at 6 months, increasing to 34% at 5 years. The authors concluded that the majority of patients undergoing TIF showed a sustained response with long-term symptom control and strong patient satisfaction [55].

Another major study comparing TIF and PPIs is the Randomized EsophyX versus Sham-Placebo Controlled TIF Trial (RESPECT). In this prospective, single-blind trial, 129 patients were randomized to either TIF 2.0 followed by 6 months of placebo or a sham endoscopy with 6 months of omeprazole therapy at maximum dosage. At the end of the study, TIF eliminated regurgitation in a larger proportion of patients than PPIs (67% vs. 47%, *p* = 0.023), and esophageal pH monitoring improved only in the TIF group (9.3% before the procedure vs. 6.3% after the procedure, *p* = 0.001) [56].

In 2017, Huang et al. published a meta-analysis on a total of 18 studies between 2007 and 2015, including 5 randomized controlled trials and 13 prospective observational studies. Compared with PPI or sham procedures, TIF showed a higher response rate (pooled relative risk 2.44, 95% CI 1.25–4.79, *p* = 0.0009) with a reduction in total reflux numbers even without significant improvement in AET. During the long-term follow-up, patients often needed to resume PPIs at reduced doses, showing a decreased efficacy of the procedure with time [52].

Despite the growing number of recent publications, the efficacy of TIF 2.0 in refractory GERD has scarcely been studied. In 2011, a prospective Italian study enrolled 20 patients with persistent heartburn and regurgitation after at least 4 weeks of high-dose therapy: 10 chose to undergo TIF 2.0 and 10 LARS. Patients were asked to perform pH impedance monitoring on PPIs before intervention and off PPIs 3 months after the intervention. The AET was normal in 100% of cases after LARS but in only 50% after TIF (*p* = 0.033). Similarly, the number of both distal and proximal refluxes was more frequently normal in the surgical than in the endoscopic group (90% vs. 20%, *p* = 0.005 and 100% vs. 40% *p* = 0.011, respectively). Persisting positive SI and SAP were found in 6 out of 10 patients in the TIF group but not in the LARS group. According to this study, despite limitations related to the small sample size and absence of randomization, EsophyX seemed to be significantly less effective than LARS in refractory GERD [57].

The efficacy of TIF on atypical GERD symptoms has been investigated by a recent meta-analysis, including 10 retrospective and prospective studies containing data from 564 patients. In all studies, the mean RSI decreased under the normality threshold 6 and 12 months after TIF. More than two thirds of patients showed persistent satisfaction with their health outcomes after the procedure [45]. 

In the longest-running cohort study to date, Testoni et al. described stable and permanent TIF outcomes regarding regurgitation and PPI use in a small cohort (14 patients) followed for up to 10 years [58].

#### 5.2.2. cTIF

TIF alone is not suitable for crural diaphragm repair but can play a role in combination with a surgical approach. Concomitant TIF (cTIF) was approved by the FDA in 2017 and consists of combined laparoscopic hernia repair and TIF for patients with hiatal hernia larger than 2 cm. Laparoscopic repair of the hiatal defect alone avoids the more extensive LARS dissection and allows subsequent execution of TIF, achieving a reduction in the prevalence of gas-bloat syndrome as compared to LARS. 

Compared to Nissen fundoplication, cTIF resulted in shorter hospital stays (1 day vs. 2 days) and a lower 30-day readmission rate (0 vs. 4.3%) without SAEs (0 vs. 4.3%). At the 6-month evaluation, minor postoperative bloating was shown with cTIF (13.8% vs. 30.0%, *p* = 0.009), but the rates of postoperative dysphagia were comparable. At the endoscopic assessment, wrap disruption and hiatal hernia recurrence were found to be equally frequent among the two groups. However, redo surgery after cTIF appears to be feasible without a significant increase in surgical morbidity, as opposed to redo fundoplication after primary Nissen [59].

#### 5.2.3. Extended Areas of Interest for TIF

As ongoing published data reinforce the evidence for TIF efficacy, the field of interest seems to widen beyond the validated applications to include fundoplication after per-oral endoscopic myotomy (POEM) or laparoscopic sleeve gastrectomy (LSG). Research on these expanded applications is now limited. 

Post-POEM GERD development is a relevant issue around a procedure increasingly adopted to treat achalasia. TIF may represent an endoscopic solution to post-POEM reflux symptoms for PPI refractory patients. A case series has been published on five patients, with 100% technical and clinical success [60]. 

LSG performed in obese patients also shows a high incidence of GERD. Since LSG is still technically feasible after a TIF procedure, TIF could be considered prior to surgery to reduce post-operative reflux symptoms [61].

### 5.3. Anti-Reflux Mucosal Interventions (ARMI)

Anti-reflux mucosal interventions (ARMI) represent the newest proposed endoluminal treatments and include anti-reflux mucosectomy (ARMS) and anti-reflux mucosal ablation (ARMA). Their aim is to, respectively, resect or ablate the mucosa at the cardia to induce a scarring process, resulting in a tightening of the gastroesophageal flap valve. As resection and ablation techniques are routinely adopted in therapeutic endoscopy, ARMI seems not to require either specific technical expertise or additional equipment, offering an attractive treatment option for GERD with a simplified learning curve and limited costs.

#### 5.3.1. Anti-Reflux Mucosectomy (ARMS)

Inoue et al. pioneered the ARMS technique in 2014. The idea originated from observing reflux symptom improvement and maintenance over time in a patient who had undergone circumferential mucosal resection for Barrett’s esophagus with high-grade dysplasia about 10 years earlier [62]. 

When first described, the ARMS procedure consisted of the resection of about two thirds of the mucosa at the EGJ along the lesser curvature of the stomach, performed in retroflexed view tracing a semicircle (“crescentic” shape) and for a total length of 3 cm (1 cm in the esophagus and 2 cm in the stomach). A 1 to 2 cm wide area was spared at the great curvature to keep the angle of His intact [63]. The treated area had previously been marked using a Dual Knife (Olympus, Tokyo, Japan) in soft coagulation mode (50 W, effect 3) in the esophagus and in forced coagulation mode (30 W, effect 3) in the stomach. Submucosal injection was carried out before resection. Different resection techniques were adopted: piecemeal mucosal resection (EMR), cap-assisted mucosal resection (EMR-C), and submucosal dissection (ESD). For ESD, a Dual Knife or ITknife nano (Olympus, Tokyo, Japan) were used in forced coagulation mode (40 W, effect 3).

The healing process of the consequent artificial ulcer resulted in the remodeling and narrowing of the flap valve in follow-up endoscopy. In this pilot study, Inoue et al. described no intraprocedural complications of ARMS performed in 10 patients with PPI refractory GERD and no sliding hernia. Nonetheless, the initial two patients who had undergone circumferential ARMS developed a significant stricture at the cardia, requiring repeated endoscopic dilations. The paper confirmed the safety of the procedure in expert hands and high clinical potential benefits, but the data were insufficient to make any recommendations. In 2017, the same group published a series of updates, including a total of 67 consecutive cases, reinforcing their initial findings. They showed a statistically significant downgrading of Hill classification in follow-up endoscopy, reduction in symptom score at 2 months (F scale from 26.8 to 8.3, *p* < 0.01; GerdQ score from 9.9 to 5.7, *p* < 0.01), and AET improvement (from 22.8% to 7.0%, *p* < 0.05). PPIs were stopped or de-escalated in 55% and 23% of patients, respectively, at 2 months, and similar results were confirmed at the 1-year follow-up [64]. 

Since this first appearance in the literature, a few modifications of the original technique have been carried out aimed at increasing safety, mostly to prevent the development of a pathological stricture at the cardia. The resection has been modified from a “crescentic” (270-degree resection) to a “butterfly” shape (180-degree resection), with the sparing of a small amount of mucosa at both the greater and the lesser curvature of the stomach. Limiting the range of mucosal resection from 270 degrees to 180 degrees has been demonstrated to reduce patients’ complaints about new-onset dysphagia without affecting either the tightness and robustness of the newly created anti-reflux barrier or symptomatic improvement [65]. The development of transient stenosis is a common finding 2–3 weeks after the procedure, but only 14.4% of patients treated with the “crescentic” resection and 5% of patients treated with the “butterfly” resection required repeated balloon dilation [66,67]. Additionally, avoiding the resection of the squamous mucosa of the esophagus has been encouraged to minimize the stricture rate [68].

Currently, the ARM-C seems to be the most widespread approach, while ESD is no longer encouraged as it can be highly technically demanding and time-consuming with a higher rate of adverse events [66,69]. Another commonly adopted variation of EMR is ligation-assisted EMR (ARM-B), consisting of the deployment of about four bands along three quarters of the EGJ after submucosal injection. The captured mucosa can then be resected with a diathermic hexagonal snare, or the bands can be left in place until they drop off spontaneously [70,71]. 

Available data on ARMS were summarized by Garg Rajat et al. in a meta-analysis published last year, including 10 studies and a total of 307 patients with refractory GERD. They reported an overall 97.7% technical success rate, with comparable outcomes regardless of the adopted technique (98.1% for EMR vs. 96.8% for EMR-B). A 17.2% rate of adverse events was described, the most common being bleeding (5%), followed by perforation, muscle injury, and aspiration pneumonia. The rate of dysphagia, secondary to the development of a stricture at the EGJ, was 11.4%, and it was influenced by the extent of resection, according to variations of the technique mentioned above. At 1 to 12 months’ follow-up, up to 65.3% of treated patients discontinued PPI usage and an adjunctive 21.5% diminished their dose. The reduction in self-reported symptoms as assessed using the GERD-HRQL or GERD-Q scale reached statistical significance. The mean AET decreased significantly by 2.39%, and the DeMeester score showed a trend toward improvement, even though it did not reach statistical significance [72].

A prospective study published in 2020 demonstrated a statistically significant increment in integrated relaxation pressure (IRP) and LES resting pressure after treatment. EGJ distensibility decreased, remaining within the normal range (from 19.0 to 13.9, *p* < 0.001). Interestingly, the procedure’s benefits were also shown in patients previously diagnosed with RH [69]. 

ARMS outcomes have been found to be similar to those of LARS, according to a recent retrospective study. Wong et al. compared 33 patients who underwent ARMS to a similar group of 67 patients who underwent Nissen fundoplication. The quality of life measured using the GERD-HRQL and RSI were comparable, and the majority of patients (about 90%) were able to discontinue PPI usage in both groups. With no differences in SAEs, ARMS had better perioperative outcomes in terms of operative time and hospital stay, pain at discharge, and the need for analgesics. Dysphagia was a significant complaint at 3 weeks postoperatively in both two groups, but ARMS performed better in terms of symptoms of gas and bloating at all scheduled follow-ups (*p* < 0.05). Unfortunately, 10 patients (30.3%) in the ARMS group required additional LARS due to persistent reflux symptoms. A major limitation of the study is the absence of objective postoperative data [68]. 

Similar results have been confirmed by another study comparing ARMS to Nissen and Toupet fundoplications. Endoscopy showed the shortest median operative time (less than 40 min vs. about 2 h for surgery) and fastest recovery (1 day vs. 5 days), with no differences in complication rate. The quality of life was similar across all groups at up to 5 years. These findings, however, could have been biased by the heterogeneity of the population (demographic, hiatal hernia type, and motility pattern differences) across the different procedures [73].

#### 5.3.2. Anti-Reflux Mucosal Ablation (ARMA)

The ARMA technique was introduced in 2020 with the purpose of inducing scarring of the EGJ through the use of mucosal ablation.

First conceived as a rescue treatment after ARMS failure, Inoue et al. performed ARMA using a TriangleTip knife J (Olympus, Tokyo, Japan) in spray coagulation mode (50 W, effect 2). Prior to treatment, a submucosal injection was used to reduce the risk of thermal injury and perforation. Mucosal ablation was performed around the cardia in a “butterfly” shape 1.5 cm wide on the gastric side, deep enough to expose the submucosal layer. Enrolled patients suffered from chronic typical GERD symptoms non-responsive to double-dose PPI treatment for at least 6 months. They had pathological 24 h pH impedance off therapy and an endoscopic Hill grade of II or III with or without erosive esophagitis grade A or B. The exclusion criteria were Hill grade IV and a large sliding hiatal hernia (>3 cm) as evaluated at upper endoscopy, primary esophageal motility disorders confirmed at HRM, age <20 years, and pregnancy. Technical success was achieved in 100% of cases (n. 12) with no immediate adverse events. At follow-up, one patient developed EGJ stricture, successfully managed with pneumatic dilation. At 2 months, all patients reported symptom improvement as assessed using the GERD-HRQL and FSSG, and 62% stopped PPI usage. Data of pH monitoring were available for eight patients only and showed a normalization of the DeMeester score in 62% of cases and a decreasing trend for AET. Changes in flap valve morphology were recorded as an improvement in Hill grade in all patients [74,75]. 

A similar technique has been described by Hernández Mondragón et al., using hybrid argon plasma coagulation therapy (Hybrid APC) to promote controlled scarring at the EGJ. Firstly, two marking lines are drawn at the great curvature with soft coagulation (40W, effect 2), spaced 1 to 1.5 cm apart, to mark the ablation area. After the injection of saline solution with methylene blue, Hybrid APC (forced coagulation, 100W, effect 3) is applied along the EGJ (270- to 320-degree) for an extension of 3 cm on the gastric side. In this pilot study, 108 patients with typical GERD symptoms refractory to PPIs and a Hill grade of II or III were enrolled. The exclusion criteria were esophageal motility disorders, hiatal hernia, and complicated GERD. Technical success was achieved in all cases without SAEs. After the procedure, patients experienced no complaints (76%), thoracic pain (12%), odynophagia (8.3%), or abdominal pain (5.3%). Almost 25% of patients reported some grade of dysphagia for solid or semisolid food, with 50% of them (n. 14) presenting with confirmed endoscopic stricture requiring dilation. Follow-up data were reported for up to 36 months for 84 patients. The GERD-HRQL score, AET, and DeMeester score significantly decreased at 3 months and were maintained over time. More than 70% of patients discontinued PPI usage and stayed off therapy, and 28% reduced their dosage. Hill grade progressively improved to grade I in all patients [76].

The results seem to be comparable between ARMA performed with the electrosurgical knife and Hybrid APC. As the range and depth of ablation can be controlled, the risk of intraprocedural perforation and delayed stricture can be assumed to be lower than with ARMS. The possibility of repeating the procedure regardless of the presence of fibrosis from previous treatment could represent another crucial feature of this approach, as ARMA could be reproposed in the case of symptomatic recurrence after a first session or after any other treatment and still maintain a good safety profile. 

A Taiwanese group has published results on a prospectively enrolled small series of 23 patients with chronic PPI-dependent GERD, defined as patients treated with PPIs for no less than 12 months and experiencing symptomatic recurrence or worsening 2 weeks after PPI discontinuation. A total of 11 patients underwent “butterfly” shaped ARMS through ESD or piecemeal EMR, and 12 patients underwent ARMA as previously described by Inoue. All patients showed a Hill grade II or III at enrollment, with or without erosive esophagitis and positive 24 h pH impedance monitoring. Patients were excluded in the case of major comorbidities or malignancies, pregnancy or lactation, >3 cm sliding hiatal hernia, upper GI varices, or major esophageal motility disorders. The median procedure time was below an hour. No major complications were described (two cases of self-limiting dysphagia and one of fever). All patients were put on PPIs for the first 3 months after the procedure, then these were stopped to observe the possible response to ARMI. After 3 months, AET improved in 83% of cases, and the reflux number was reduced in almost 78% of cases. About 74% of patients reported subjective global improvement, and 57% were off PPIs. Interestingly, controversial results were found in a subgroup of patients: one (4.3%) had worsened erosive esophagitis and reflux symptoms, and three (13%) showed an increased AET. In these patients, an endoscopic evaluation confirmed no or only partial improvement in the flap valve. The study concluded that ARMI is generally effective and safe in PPI-dependent patients with promising results in terms of AET improvement and PPI discontinuation rate. However, the negative effects of ARMI on some patients should be carefully observed. Objective testing after ARMI is necessary to avoid PPI discontinuation in patients who can present with improved global symptoms but worsening acid exposure [77].

#### 5.3.3. Extended Areas of Interest for ARMI

With regard to TIF, initial research is expanding through a more complex area of application of ARMI, including post-POEM and post-LSG patients.

Hernández Mondragón et al. published preliminary results on ARMA applied to a small cohort of post-POEM patients with objective evidence of GERD. Satisfying symptomatic control was achieved in five patients out of six [78]. 

Debourdeau et al. showed the feasibility of ARM-B for treating GERD after LSG as an alternative to surgery. Despite challenges originating from the altered post-surgical anatomy, six procedures were carried out, with five cases of clinical success. One patient developed an esophageal stricture, which was successfully managed endoscopically [79].

Clearly, the results are too limited both in terms of sample size and follow-up to provide any advice for clinical practice.

## 6. Discussion

GERD is a gastrointestinal disorder with a high prevalence worldwide among all age and gender groups and brings a relevant physical, mental, and socio-economic impact. Recent years have seen a progressive evolution in GERD diagnosis and treatment along with a better understanding of its physiopathology.

Nonetheless, the heterogeneity in GERD spectrum manifestations can make effective treatment challenging. The first-line therapeutic strategy of lifestyle changes and antisecretory medications can be effective for most patients, who can be confidently managed in primary care. 

Nevertheless, a not negligible portion of patients experience suboptimal response to PPIs and frequent symptomatic relapse when medications are stopped. Finally, younger patients unwilling to take lifelong medications or those worried about their potential long-term adverse effects also deserve a personalized management strategy.

LARS is a well-established procedure and represents the standard of care when medical therapy fails. However, cautiousness is common among surgeons, and patients are frequently reluctant to proceed to surgery. The number of LARS procedures has been declining over the last decade; currently, in the USA, fewer than 30,000 Nissen fundoplication procedures are performed per year, meaning that less than 1% of GERD patients are referred to surgery. Controversy around surgical approach has arisen because of the risk of adverse events compared to medical treatment and the uncertainty around long-term efficacy. Symptoms of dysphagia, gas-bloat syndrome, and bowel dysfunctions have been described in one out of four treated patients. Experienced surgeons in high-volume centers report long-term symptom recurrence in only 10% to 15% of patients, but the results are more variable in lower-volume community hospitals; while up to 90% of patients experience relief from GERD symptoms in the short term, the need to resume medical therapy gradually increase to encompass 25% to 62% of patients at 5 to 15 years after surgery [37,38,39,40]. 

Throughout the last two decades, endoscopists have attempted to fill the treatment gap through the development of attractive minimally invasive alternative techniques. However, endoscopic procedures’ diffusion is still restricted to a few tertiary centers worldwide. 

Different devices have been introduced to offer the possibility of performing an endoscopic fundoplication as close as possible to the surgical technique. We mainly discussed TIF with regard to EsophyX, as it has shown the most promising data. 

Since its introduction in 2007, over 27,000 TIF procedures have been performed with the EsophyX device. The available studies have concluded that there is good postoperative long-term symptom control, with an attained responsive rate of 65% at 6 months. Patients’ global satisfaction with their health outcomes ranges from 45 to 86% at a mean of 6 months, despite the frequent need to resume PPI usage at some point; the discontinuation of PPI usage gradually falls from 70% at 6 months to about 30% at 5 years. Nevertheless, about 80% of patients will eventually need a lower dosage compared to pre-endoscopy assessment [45,52,53,54,55,56,58]. The TIF procedure has also shown an overall good safety profile, with common mild and transient postoperative complications and few SAEs, mostly managed conservatively (around 2%) [50,51,52]. However, as it requires additional single-use equipment, TIF can be costly and presents a steep learning curve, even for experienced endoscopists. The high success rate and safety profile of the procedure has to be confirmed outside of referral centers, as results can be biased by operator expertise. ARMS and, most recently, ARMA are gaining increasing attention, especially due to their simplicity and low risk of complications, and because they do not require costly add-on devices. Based on current knowledge, it has been hypothesized that scar formation at the EGJ may work by reducing the backflow volume of gastric content as a result of reduced distensibility; it is also reasonable to suppose a decrease in chemo- and mechano-receptor sensitivity to acid exposure. ARMI can be performed in an ambulatory setting and, because of a simplified learning curve, can be more easily reproduced among a wider number of endoscopists. ARMA could also be considered an option for additional treatment when partial recurrence occurs after previous surgical or endoscopic treatment. The published data suggest the efficacy of ARMI in more than 60% of treated patients, mainly in terms of subjective perception and the tapering or discontinuation of PPI usage. Follow-up data on objective testing (endoscopy, HRM, and pH impedance) are very restricted, but, when reported, they show improvement in LES parameters (Hill grade, IRP, and LES resting pressure) and a significant reduction in AET after treatment. New-onset dysphagia is infrequent (a rate of about 5%) and no SAEs have been described. No data are currently available regarding long-term durability [64,69,72,74,75,76,77].

According to retrospective studies comparing endoscopic options to surgery, endoscopy performs similarly in terms of symptom control and quality of life improvement, with comparable adverse event rates and the advantage of a shorter operative time and better postoperative recovery [59,68,73].

However appealing, these results should be treated with caution. The major limitations of endoscopic treatments should be taken into account, primarily related to the heterogeneity of the GERD population and the lack of standardization of procedures; the results of the available studies have mainly focused on symptomatic improvement based on patients’ reports or reduction in PPI consumption after treatment. In fact, few trials provide precise results of post-treatment diagnostic tests, and, when available, objective outcomes are largely partial. Additionally, the sample size is often small, and follow-up is variable between studies. 

Currently, anti-reflux endoscopic treatments are not endorsed by the European Society of Gastrointestinal Endoscopy (ESGE), which advised against their routine use in their 2020 guidelines due to limited data on the long-term outcomes and potential risks [80]. By contrast, despite the low quality of evidence, the American College of Gastroenterology (ACG) 2022 clinical guidelines suggested consideration of TIF for patients with troublesome regurgitation or heartburn who do not wish to undergo surgery and do not have severe esophagitis or hiatal hernias > 2 cm [7].

In our view, endoscopic treatments should not be considered a substitute for surgery, but both approaches should be integrated into clinical practice based on the characteristics of each patient in order to address the majority of the GERD population. ARMI could be adopted as a first-line procedure to treat long-term recurrent GERD symptoms and reduce PPI consumption in patients with proven mechanical incompetence of the LES and no other major anatomic disruption, Esophyx could be used in the presence of a small hiatal hernia when reestablishing an intraabdominal esophageal segment is needed, and surgery could be reserved for the correction of considerable diaphragmatic crural defects as in large hiatal hernia.

## 7. Conclusions

Tailor-made strategies are key to both the successful treatment of GERD chronic and recurrent symptoms and the prevention of its possible long-term complications. Specifically, interventional endoscopy has great potential in the near-future landscape.

However, the successful spread of endoluminal anti-reflux techniques requires more studies following strict inclusion criteria, standardization of procedures, and objective outcomes. The currently available findings are encouraging, but we still need validation from prospective studies, larger cohorts, and longer follow-ups.

## Data Availability

Not applicable.
